# Sudden unexpected death in early childhood: general observations in a series of 151 cases: Part 1 of the investigations of the San Diego SUDC Research Project

**DOI:** 10.1007/s12024-015-9724-2

**Published:** 2016-01-19

**Authors:** Marco M. Hefti, Hannah C. Kinney, Jane B. Cryan, Elisabeth A. Haas, Amy E. Chadwick, Laura A. Crandall, Felicia L. Trachtenberg, Dawna D. Armstrong, Marjorie Grafe, Henry F. Krous

**Affiliations:** Department of Pathology, Boston Children’s Hospital, Boston, MA USA; Department of Pathology, Beth Israel Deaconess Medical Center, Boston, MA USA; Department of Pathology, Rady Children’s Hospital-San Diego, San Diego, CA USA; The SUDC Foundation, Hackensack, NJ USA; New England Research Institutes, Watertown, MA USA; Department of Pathology, Emeritus, Baylor College of Medicine, Houston, TX USA; Department of Pathology, Oregon Health and Science University, Portland, OR USA; Department of Pediatrics, UCSD School of Medicine, La Jolla, CA USA; Division of Neuropathology, Department of Pathology, Mount Sinai School of Medicine, One Gustave L. Levy Place, New York, NY 10029 USA; Division of Neuropathology, Beaumont Hospital, Beaumont Road, Dublin 9, Ireland; Department of Neurology, NYU Langone Medical Center, New York, NY USA

**Keywords:** Sudden unexpected death in childhood, Sudden death, Febrile seizures, Hippocampus

## Abstract

**Purpose:**

The purpose of this study was to determine the major subcategories and clinicopathologic features of sudden unexpected death in young children in a large retrospective cohort, and to confirm the association of sudden unexplained death in children (abbreviated by us for unexplained deaths as SUDC) with hippocampal pathology and/or febrile seizures.

**Methods:**

We undertook analysis of a retrospective cohort of 151 cases, of which 80 % (121/151) were subclassified as SUDC, 11 % (16/151) as explained, 7 % (10/151) as undetermined, and 3 % (4/151) as seizure-related.

**Results:**

There were no significant differences between SUDC and explained cases in postnatal, gestational, or postconceptional age, frequency of preterm birth, gender, race, or organ weights. In contrast, 96.7 % (117/121) of the SUDC group were discovered during a sleep period compared to 53.3 % (8/15) of the explained group (*p* < 0.001), and 48.8 % (59/121) of the SUDC cases had a personal and/or family history of febrile seizures compared to 6.7 % (1/15) of the explained group (*p* < 0.001). Of the explained deaths, 56 % (9/16) were subclassified as infection, 31 % (5/16) cardiac, 6 % (1/16) accidental, and 6 % (1/16) metabolic. Two of the three cases specifically tested for cardiac channelopathies at autopsy based upon clinical indications had genetic variants in cardiac genes, one of uncertain significance. Bacterial cultures at autopsy typically revealed organisms interpreted as contaminants. Two of the four seizure-related deaths were witnessed, with two of the brains from these cases showing generalized malformations. Hippocampal anomalies, including a specific combination we termed hippocampal maldevelopment associated with sudden death, were found in almost 50 % (40/83) of the SUDC and undetermined cases in which hippocampal sections were available.

**Conclusions:**

This study highlights the key role for the hippocampus, febrile seizures, and sleep in SUDC pathophysiology. It also demonstrates the role of known predisposing conditions such as cardiac channelopathies and infections in causing sudden unexpected death in childhood, and the need for improved ancillary testing and protective strategies in these cases, even when the cause of death is established at autopsy.

## Introduction

Sudden unexplained death in childhood (SUDC) is the sudden and unexpected death of a child older than 1 postnatal year that remains unexplained after a review of the clinical history and circumstances of death, and the performance of a complete autopsy [1]. It is a subset of sudden and unexpected death—the subset without explanation. While a rare disorder (with an incidence of 1.3/100,000 between 1 and 4 years), SUDC nevertheless accounts for approximately 10 % of all unexpected childhood deaths in population-based studies although by definition, the rate will vary depending on the completeness of the investigation undertaken [2–5]. Our overall hypothesis is that SUDC is comprised of a heterogeneous group of diseases yet to be discovered that share the clinical phenotype of sudden unexpected death in a seemingly healthy child. In 1999, the San Diego SUDC Research Project was founded to attempt to discover the causes of these deaths through in-depth review of retrospectively accrued cases via national and international referrals to a centralized database [6, 7]. While such a dataset is limited by its retrospective, non-population based design, in an initial study we were nevertheless able to identify a subset of SUDC cases with multiple structural abnormalities of the hippocampus with or without a personal and/or family history of febrile series [8]. In this study, we report the findings of the entire database from 1999 to 2011, which includes an addition of 87 cases of sudden unexpected death including 72 additional unexplained (SUDC) cases.

The main goal of the present study was to determine the major subcategories and clinicopathologic features of sudden unexpected death in young children in the entire complete San Diego cohort of 151 cases, the largest such series to date. Our second objective was to confirm the association of SUDC with hippocampal pathology and/or febrile seizures found in the initial analysis of the dataset in the expanded cohort. Due to the large scope of the findings, the neuropathology of the hippocampus and other brain regions in SUDC associated with hippocampal maldevelopment is reported in detail in a separate publication.

## Materials and methods

### Clinicopathologic dataset of San Diego SUDC research project

The cases of sudden unexpected death in this study were accrued under the auspices of the SUDC Research Project from 1999 to 2011. We have previously described the mission, history, design, and methods of the study in detail [6–9]. The dataset was based mainly upon case referrals from the SUDC Program, Hackensack, NJ (LAC), which in turn received referrals from families, medical examiners, coroners, and pathologists nationally and internationally. The inclusion criteria were: (1) parental consent for research; (2) autopsy not restricted to a single organ; and (3) death between 1 and 15 years. This paper examines data for the major group of children who were 1–6 years (<7 years) old. There were no cases of homicide or suicide in this cohort, given that the study was based upon a referral dataset of sudden and unexpected deaths due to natural causes, particularly those that were unexplained.

For each case, autopsy reports, death scene investigations, pediatric and obstetrical records, ancillary testing (e.g., toxicology, metabolic screening), and any available glass microscopic slides of the systemic organs and brain, were collected for review. In a small number of cases it was possible to obtain formalin-fixed tissues from which additional slides were prepared and reviewed. In addition, the majority of parents completed a detailed study questionnaire concerning the child’s medical, neurodevelopmental, environmental, and family histories. In-depth telephone conversations were undertaken with the parents or caretakers to verify and/or clarify information in the family surveys (HFK, LAC). The Institutional Review Boards of Rady Children’s Hospital, San Diego, CA, and Boston Children’s Hospital, Boston, MA.

### Definitions of cause of death categories

We classified the sudden and unexpected deaths into four categories: SUDC, explained, undetermined, and (non-febrile) seizure-related disorder. SUDC was defined as above [1]. Explained deaths were those in which postmortem studies revealed a known cause of death, including natural diseases and accidental trauma. Explained cases in which there was a history of at least one witnessed, non-febrile seizure, or epilepsy (defined as two or more non-febrile seizures), were classified as a separate “seizure-related” category due to the known hippocampal pathology of epilepsy. In contrast, febrile seizures were defined as convulsions associated with fever in the absence of central nervous system infection [10]. Undetermined deaths were those in which pathologic and/or death scene findings were identified, but their contribution to the cause of death could not be determined with a high level of certainty [6–8]. The team’s pediatric pathologist (HFK) in consultation with the pediatric neuropathologists (HCK, MG) performed the adjudication.

### Neuropathologic review of the autopsy

**Fig. 1:**
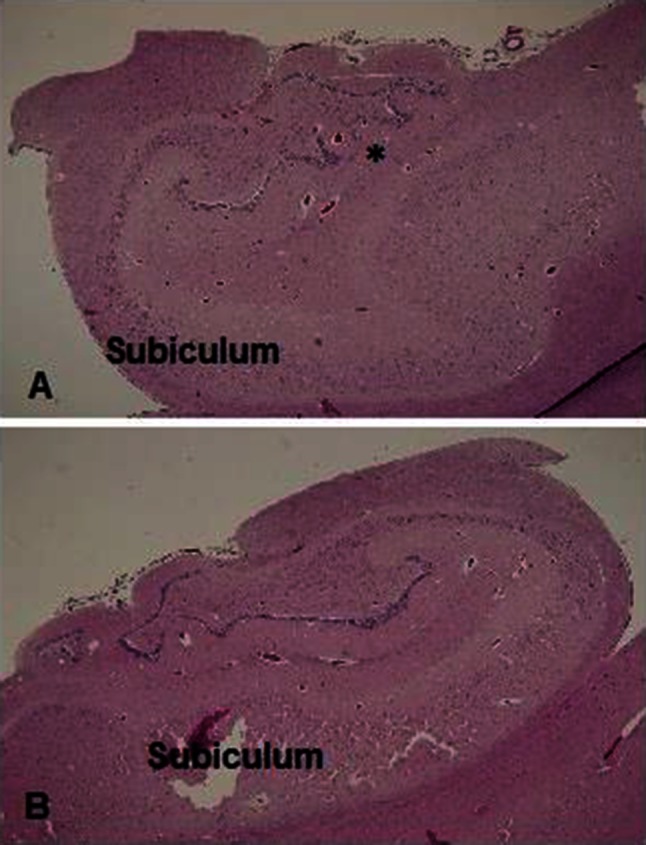
The left and right hippocampi are asymmetric. On Side **a**, the subiculum is abnormally thick and slightly folded compared to the contralateral side **b**. The dentate gyrus is hyperconvoluted bilaterally, but dissimilar in shape. On Side **a** the dentate gyrus encircles a blood vessel in the molecular layer (*asterisk*) and is partially duplicated

The study’s neuropathology group (HCK, MG, JC, DDA, MH) all reviewed the neuropathology reports and microscopic sections of the brain and spinal cord (when available) in concert with the available clinicopathologic data. A separate, blinded, review of the hippocampal sections was undertaken by two of the authors (HCK, MMH) in the cases that had them, looking for the same features identified in our previous publications [8, 9]. These features included focal granule cell bilamination (partial duplication) of the dentate gyrus (Fig. 1), hyperconvolution of the dentate gyrus, heterotopia in the hippocampus or adjacent temporal regions, single or clusters of granule cells in the molecular layer of the dentate gyrus, malrotation and/or asymmetry (Fig. 1) of the hippocampus, anomalies of the subiculum and/or entorhinal cortex, vertical layering of the temporal cortex, and qualitatively increased interstitial neurons [8, 9]. Given the well-recognized variation in cellular architecture of the human hippocampus throughout its structure, and variation in sectioning between different medical examiner and coroner’s offices, inclusion in the study required the sections be oriented properly in the coronal plane, as identified by adjacent anatomic landmarks such as the lateral geniculate nucleus and tail of the caudate, and in comparison to normative human atlases of the hippocampus [11]. Cases without proper orientation of the dentate gyrus in the hippocampal section, or that were technically unsatisfactory (fold, tears), were excluded from analysis, as were sections consisting entirely of pes. The hippocampal sections, 6–10 microns thick, were all stained with hematoxylin-and-eosin or hematoxylin-and-eosin with Luxol-fast-blue counter stain, and examined with a standard light microscope. It was not possible to obtain additional sections for immunohistochemical or other ancillary studies across the medical examiner systems from which cases were accrued, and thus, these could not be performed in this study.

### Supplementation of autopsied cases of young children 1–6 years old for determination of the neuropathology of hippocampal pathology

Due to the small number of explained cases with hippocampal sections, we reviewed the autopsy archives of Boston Children’s Hospital (BCH) during the years covered by this study to identify additional control cases. These cases met the same criteria described above with the exception that the deaths were explained, and neither sudden nor unexpected. They were reviewed in the same manner as the San Diego cases. These cases were used only in the analysis of hippocampal features described below.

### Statistical analysis

Comparisons were made using *t* tests for means, Wilcoxon rank sum tests for medians, analysis of covariance controlling for age for organ weights, or Chi square tests for frequencies. All analyses were conducted using SAS v9.3 (SAS Institute Inc., Cary, NC), and statistical significance was tested at a level of 0.05.

## Results

### Overview of total cohort of sudden unexpected death in the San Diego Cohort

A total of 151 cases of sudden unexpected death in children between 1 and 6 years of age (<7 years) were available for analysis. Of these 151 cases, we classified 80 % (121/151) as SUDC, 11 % (16/151) as explained, 7 % (10/151) as undetermined, and 3 % (4/151) as (non-febrile) seizure-related sudden death (Table 1). There were no statistically significant differences between SUDC and explained cases in postnatal, gestational, or postconceptional age, or the frequency of preterm birth, gender, or race (Table 1). The most striking difference between the SUDC and explained categories (not including cases with non-febrile seizure disorders) was the frequency in which the deaths were discovered during a sleep period: 96.7 % (117/121) of the SUDC cases compared to 53.3 % (8/15) of the explained cases (*p* < 0.001) (Table 1). The two other distinguishing features between the SUDC and explained categories related to febrile seizures: 31.4 % (38/121) of the SUDC cases had a personal history of febrile seizures compared to none (0/16) of the explained group (*p* = 0.01). Moreover, 48.8 % (59/121) of the SUDC cases had a personal and/or family history of febrile seizures compared to 6.7 % (1/15) of the explained group (*p* < 0.001) (Table 1). Thus, we confirmed the association of SUDC with a personal and/or family history of febrile seizures, as in the initial cohort [7–9].

**Table 1:** Clinicopathologic features in four categories of sudden unexpected death in children (1–6 postnatal years) in the expanded San Diego Cohort (*n* = 151) according to groups of explained, seizure-related, undetermined, and SUDC

	Explained	Seizure-Related	Undetermined	SUDC	*p* value SUDC versus explained
	*n* = 16 (10.6 %)	*n* = 4 (2.6 %)	*n* = 10 (6.6 %)	*n* = 121 (80.1 %)	
*Demographics*					
Age at death (years)	1.61, 1–5.62	1.91, 1.52–3.88	1.67, 1.01–6.59	1.67, 1.02–6.72	NS
Gestational age (weeks)	39.22 ± 1.56 (*N* = 15)	37.93 ± 1.08	37.7 ± 4.92	38.76 ± 2.42 (*N* = 114)	NS
Preterm birth^a^	12.5 % (2/16)	0 % (0/4)	10 % (1/10)	11.9 % (14/118)	NS
Male gender	62.5 % (10/16)	50 % (2/4)	70 % (7/10)	62 % (75/121)	NS
White race	87.5 % (14/16)	100 % (4/4)	100 % (10/10)	79.3 % (96/121)	NS
Personal history of febrile seizures	0/16 (0 %)	1/4 (25 %)	1/10 (10 %)	38/121 (31.4 %)	0.01
Family history of febrile seizures	1/15 (6.7 %)	2/3 (66.7 %)	3/9 (33.3 %)	38/121 (31.4 %)	0.07
Personal and/or family history of febrile seizures	1/15 (6.7 %)	2/4 (50 %)	3/9 (33.3 %)	59/121 (48.8 %)	<0.001
*Circumstances of death*					
Fever within 48 h of death	10/16 (62.5 %)	2/3 (66.7 %)	5/10 (50 %)	61/120 (50.8 %)	NS
Head trauma within 48 of death	1/13 (7.7 %)	0/2 (0 %)	1/5 (20 %)	11/63 (17.5 %)	NS
Death during a sleep period (overnight or nap)	8/15 (53.3 %)	2/4 (50 %)	9/10 (90 %)	117/121 (96.7 %)	<0.001
Sleep position					NS^b^
Found prone	6/10 (60 %)	0/2 (0 %)	6/9 (66.7 %)	96/114 (84.2 %)	
Found on side	1/10 (10 %)	0/2 (0 %)	1/9 (11.1 %)	13/114 (11.4 %)	
Found supine	1/10 (10 %)	1/2 (50 %)	2/9 (22.2 %)	4/114 (3.5 %)	
Found in other position	2/10 (20 %)	1/2 (50 %)	0/9 (0 %)	1/114 (0.9 %)	
Found face-down	3/9 (33.3 %)	0/2 (0 %)	6/8 (75 %)	59/108 (54.6 %)	NS
Season of death					NS^c^
Winter	4/16 (25 %)	1/4 (25 %)	2/10 (20 %)	41/121 (33.9 %)	
Spring	3/16 (18.8 %)	0/4 (0 %)	5/10 (50 %)	36/121 (29.8 %)	
Summer	2/16 (12.5 %)	0/4 (0 %)	2/10 (20 %)	23/121 (19 %)	
Fall	7/16 (43.8 %)	3/4 (75 %)	1/10 (10 %)	19/121 (15.7 %)	
Death at home	11/16 (68.8 %)	1/4 (25 %)	7/10 (70 %)	100/121 (82.6 %)	NS
Number of ER visits prior to death	0 ± 0 (*N* = 2)	1 ± 1.41 (*N* = 2)	2 ± . (*N* = 1)	1.5 ± 1.06 (*N* = 40)	<0.001
*Autopsy*					
Tongue laceration	0/5 (0 %)	(*N* = 0)	1/6 (16.7 %)	3/57 (5.3 %)	NS
Pulmonary edema	6/15 (40 %)	2/4 (50 %)	2/8 (25 %)	48/115 (41.7 %)	NS
Focal pulmonary hemorrhage	3/15 (20 %)	0/3 (0 %)	1/9 (11.1 %)	16/116 (13.8 %)	NS
Brain weight (g)	1205.4 ± 37.27 (*N* = 16)	1223.84 ± 74.29	1217.77 ± 46.97	1225.12 ± 13.64 (*N* = 119)	NS
Head circumference (cm)	49.17 ± 0.87 (*N* = 6)	49.09 ± 1.53 (*N* = 2)	48.88 ± 0.81 (*N* = 7)	49.07 ± 0.26 (*N* = 68)	NS
Thymus weight (g)	42.06 ± 3.94 (*N* = 13)	22.36 ± . (*N* = 1)	29.42 ± 4.72 (*N* = 9)	35.01 ± 1.37 (*N* = 107)	NS
Heart weight (g)	69.66 ± 3.63 (*N* = 15)	63.94 ± 7.04	61.29 ± 4.69 (*N* = 9)	65.74 ± 1.31 (*N* = 116)	NS

Mean ± SD with *t* test or median, range with Wilcoxon test, or *n*/N (%) with Fisher exact test. Organ weights and head circumference are age-adjusted with age-adjusted mean ± SE, ANCOVA

^a^<37 weeks gestation

^b^ *p* value for prone versus side versus supine

^c^ *p* value for winter versus spring versus summer versus fall

There were no significant differences in history of head trauma or fever within 48 h of death, sleep position at discovery (where applicable), season of death, or location of death (home vs. other, e.g., day care) among the SUDC and explained groups (Table 1). There were also no significant differences in organ weights (adjusted for age), or any other general autopsy findings (Table 1). As part of the data accrual for the SUDC Research Project, we collected socioeconomic data on the parents of each child. Eighty-nine percent (134/151) of the families in the San Diego cohort provided information about yearly household income, with the following results: 80 % (107/137) > $50,000; 17 % (16/137) $10,000–50,000, and 3 % (4/137) < $10,000. Eighty-five percent of both the mothers and the fathers completed college and/or postgraduate education. There were no significant associations between level of annual income or education among the SUDC, explained, seizure-related, or undetermined categories.

### Ancillary testing in San Diego Cohort

The use of ancillary testing at autopsy in the San Diego cohort is summarized in Table 2. Vitreous electrolytes were done in 49 % (74/151) cases and were not contributory to identifying a cause for sudden death in any case. Postmortem radiology was performed in 43.7 % (66/151); no diagnostic observations contributing to sudden death were made. Metabolic screening for inborn errors of metabolism at autopsy was performed in 48.3 % (73/151) and was abnormal in 1.3 % (1/73). The positive case was an explained case showing elevated acylcarnitine species in postmortem bile suggestive of a disorder of fatty acid metabolism (see below). Postmortem cultures were performed in 69.5 % (105/151) of cases, and were positive in 27.6 % (29/105) of those tested; there was no substantial difference among the frequency of positive cases among the subcategories of sudden unexpected death (Table 2). The positive cultures in the SUDC group were either considered contaminants, of unknown pathogenicity, or not associated with lethal inflammatory histopathology at autopsy. Toxicology was performed in 93.5 % (143/151) cases, and was positive in 5.6 % (8/143) of those in which it was done. Substances identified included caffeine (*n* = 3), carboxyhemoglobin (*n* = 2), theobromine, nicotine (likely due to passive smoke), methemoglobin, acetone, and methadone (*n* = 1); none were considered to demonstrate lethal levels. The detection of methadone was of very low level, with no obvious sources, and was considered to be a possible laboratory false positive; it was not considered related to the child’s sudden death.

**Table 2:** Ancillary testing in sudden unexpected death in childhood by subcategory

	Explained	Undetermined	SUDC	SUDEP
	*N* = 16	*N* = 10	*N* = 121	*N* = 4
Toxicology performed	94 % (15)	100 % (10)	95 % (115)	75 % (3)
Positive	0	2	6	0
Microbiology performed	69 % (11)	50 % (5)	72 % (87)	50 % (2)
Positive	3	3	32	0
Vitreous electrolytes performed	31 % (5)	60 % (6)	51 % (62)	25 % (1)
Abnormal	0	0	0	0
Postmortem metabolic screening	56 % (9)	30 % (3)	49.5 % (60)	25 % (1)
Abnormal	1	0	0	0
Cardiac genetic testing at autopsy	13 % (2)	0 % (0)	1 % (1)	25 % (1)
Positive	1	0	0	0
Radiology for boney trauma or natural disease	31 % (5)	30 % (3)	48 % (58)	0 % (0)
Positive	0	0	0	0

The number of cases sampled for a particular ancillary test and the percent with positive results are tabulated. The microbiological cultures were predominantly from nasopharynx and lung, followed by blood

### Explained category

In the San Diego cohort explained group (*n* = 16), the mean postnatal age was 1.6 years (range 1–5.6 years), and the frequency of male gender was 62.5 %; preterm birth, 12.5 %, and Caucasian, 87.5 % (Table 2). Fifty-six percent (9/16) of the explained deaths were attributed to infection. Two of these 9 cases were due to respiratory infection, and 3 due to myocarditis, as determined by microscopic criteria (Table 3). There were also three cases of fatal neurogenic pulmonary edema secondary to aseptic meningitis, two of which we have previously described [12]. In 78 % (7/9) of the infectious cases, clinical symptoms were present prior to death, e.g., fever, headache, and general malaise; the other two cases were asymptomatic and presented as sudden death. The mean duration of symptoms in infectious cases with a clinical prodrome was 2.16 days (range 1–7). Of the 9 infectious cases, a physician evaluated only one prior to death.

**Table 3:** Clinicopathologic features in cases of explained and seizure related sudden unexpected death

#	Age	Sex	Category	Cause of Death	HMASD
1	1	M	Accident	Environmental hyperthermia in overheated bedroom with dehydration	NA
2	1.1	F	Infection	Cardiac: Myocarditis	No
3	1.3	M	Infection	Cardiac: Myocarditis	Yes
4	4.3	F	Infection	Cardiac: Myocarditis	NA
5	1.5	M	Infection	Pulmonary: Acute bronchopneumonia	NA
6	1.4	M	Infection	Pulmonary: Tracheobronchitis and bronchiolitis	No
7	5	M	Infection	Sepsis	NA
8	1.8	M	Infection	CNS: Aseptic meningitis with neurogenic pulmonary edema(10)	NA
9	1.9	M	Infection	CNS: Aseptic meningitis with neurogenic pulmonary edema(10)	No
10	2.5	M	Infection	CNS: Aseptic meningitis with neurogenic pulmonary edema(10)	NA
11	1.4	M	Metabolic	Fatty Acid Metabolic Disorder	NA
12	1.5	M	Cardiac	Congenital: William’s syndrome with coronary artery stenosis (antemortem genetic testing)(11)	No
13	1.3	F	Cardiac	Congenital: Aortic stenosis	No
14	1.8	F	Cardiac	Cardiomyopathy: Histiocytoid cardiomyopathy	NA
15	4.7	F	Cardiac	Arrhythmia: Long QT syndrome (postmortem genetic testing)	NA
16	5.5	F	Cardiac	Arrhythmia: CPVT (postmortem genetic testing)	No
17	1.5	F	Seizure-related	Single, lethal, non-febrile seizure that occurred during waking and was witnessed	NA
18	1.9	F	Seizure-related	Single, lethal, non-febrile seizure witnessed upon waking	No
19	1.9	M	Seizure-related	Cerebral palsy, failure to thrive, unspecified seizure disorder, found dead after sleep period	No
20	3.8	M	Seizure-related	Epilepsy, preceded by history of febrile seizures, developmental delay	No

*CNS* central nervous system, *M* male, *F* female, *HMASD* hippocampal malformation with sudden death, *NA* hippocampal section not available. Age is in years

The second leading category of explained deaths [31 % (5/16)] was due to primary (non-infectious) cardiac abnormalities, including congenital malformations, histiocytoid cardiomyopathy, and arrhythmia disorders (Table 3). Histiocytoid cardiomyopathy was diagnosed in one case (Case 14) at autopsy by microscopic examination. We previously reported the case of coronary artery stenosis and sudden death in the child with Williams syndrome (Case 12) whose diagnosis was confirmed by a fluorescent in situ hybridization (FISH) study prior to death that showed a heterozygous deletion of band 7q11.23 [13]. Specific testing during the child’s life was sought based upon the clinical findings suggestive of Williams syndrome. At autopsy, the brain demonstrated cerebral cortical, but not hippocampal, abnormalities. The second case of congenital heart disease (Case 13) was suspected to have mild pulmonary stenosis based on clinical examination by a pediatric cardiologist and electrocardiogram (EKG). At the time of autopsy, the child was found to have aortic stenosis with poststenotic aortic dilatation, cardiomegaly, and left ventricular hypertrophy. The two cases of genetic arrhythmia syndromes were long QT syndrome with KVLQT1 mutation (Case 15), and catecholaminergic polymorphic ventricular tachycardia (CPVT) (Case 16). The former case was initially successfully resuscitated, and died after a prolonged intensive care unit stay during which multiple EKGs showed a prolonged QT-interval. The second arrhythmia case (Case 16) had an earlier episode of enterovirus infection thought to be associated with myocarditis; electrocardiographic evaluation showed episodic, non-sustained supraventricular tachycardia and aberrant conduction, and the echocardiogram was normal. In both cases, genetic testing was performed postmortem. Cardiac genetic testing was considered in four cases at autopsy based upon premortem findings, e.g., prolonged QT interval on electrocardiograms in intensive care unit (see above), and was performed in three after thorough case review. The KVLQT1 mutation was considered pathogenic and of *de novo* origin after trio testing (Case 15). The CPVT-related mutation (Case 16) was considered a genetic variant of unknown clinical significance.

The remaining two explained deaths were due to accidental hyperthermia (Case 1), and an unspecified fatty acid metabolic disorder (Case 11), the latter diagnosis based on a fatty liver and elevated acylcarnitine species in the postmortem bile. The deaths in the BCH (*n* = 16) cases used only for the hippocampal analysis were all hospitalized and therefore anticipated and not sudden or unexpected. The postnatal age at death ranged from 1.1 to 6.0 years, with a mean of 3.3 years. There were 8 males and 8 females. The causes of death were congenital heart disease (*n* = 3), malignancy (*n* = 6), infection (*n* = 4), and non-cardiac (non-neural) congenital malformations (*n* = 3).

### Undetermined group

The undetermined group was on average 1.7 years old at death, male, full-term, and discovered in the prone position (67 %) face-down (75 %) during a sleep period (90 %) with a fever (50 %). Like SUDC cases they also had a higher rate of febrile seizures than controls, although the number of cases is too low for significant analysis (Table 1). Pathologic findings in this group included unsuspected pilocytic astrocytoma in the cerebellum, mild neuropathologic sequelae of prematurity, mild carnitine deficiency, and focal microscopic brainstem encephalitis. These findings were considered of uncertain relationship, if any, to the causal chain of events leading to sudden death, and most likely to be incidental.

### Seizure-related causes of sudden unexpected death

We consider this group of four cases to be explained, but separate it from the other causes of explained deaths in order to potentially distinguish its features from those of the SUDC cases with a personal and/or family history of febrile seizures, and in whom sudden death possibly related in some way to aberrant electric discharges or seizures. The mean postnatal age of the four non-febrile seizure related deaths was 1.9 years (range 1.5–3.9 years); the frequency of male gender was 50.0 %; preterm birth, 0 %, and Caucasian, 100.0 % (Table 2). Two cases (Cases 17 and 18) had a lethal non-febrile seizure during waking, which was witnessed by a parent (Table 2). The third case (Case 19) had cerebral palsy, microcephaly, failure to thrive, and an associated seizure disorder. The fourth case (Case 20) had severe epilepsy that developed after an initial history of febrile seizures. In all instances, sudden death was attributed to a lethal seizure, although in the third and fourth cases (Cases 19 and 20, respectively), the deaths occurred during a sleep period and were not witnessed, and sudden unexplained death in epilepsy (SUDEP) was considered (Table 3). Case 17 of the four seizure-related cases did not have a hippocampal section for evaluation; and in Cases 18 and 19, the hippocampus was without pathologic changes (Table 3). Case 20 in which the child’s epilepsy was chronically treated with anti-epileptics had a malformed brain at autopsy with medial and lateral temporal and extra-temporal anomalies, differing from the findings described below in SUDC cases. In two cases a single observed fatal seizure occurred in a child without a previous history of seizures. Based on extensive reviews of the investigative reports, medical histories, and all other available information, these appear to represent true fatal seizures rather than secondary or agonal events due to a primary systemic cause of death, but the possibility of a non-CNS mediated process cannot be completely excluded.

### Sudden unexplained death in childhood group

The SUDC group was, on average, 1.7 years old at death, male, full-term, and discovered dead in the prone position (84 %), face-down (55 %) during a sleep period (97 %) with a fever (51 %) (Table 2). Microbiologic testing at autopsy was positive in 36.8 % (32/87) of the SUDC cases where it was performed. These were polymicrobial in 53.1 % (17/32) cases, including 7 cases that were positive for both bacterial and viral species. The majority of positive cultures were from respiratory sources [65.6 % (21/32)]. In all cases, these were judged to be either contaminants, or not sufficient to warrant an infectious cause of death in light of histologic and other findings. The rate of positive cultures did not differ significantly between SUDC and explained cases (24/87 vs. 2/11, *p* = 0.72).

Fifty-five percent (83/151) of the entire cohort had at least one hippocampal section for microscopic review, with the following frequency of available hippocampal sections in each group: SUDC, 57 % (69/121), explained, 44 % (7/16), undetermined, 70 % (7/10), and seizure-related death, 75 % (3/4). A total of 96 cases (83 San Diego and 16 BCH) constituted the cohort for the hippocampal analysis below. Cases of SUDC were significantly more likely to show focal granule cell bilamination [43.5 % (30/69) vs. 13.0 % (3/23), *p* = 0.01] and gross malrotation [20.3 % (14/69) vs. 0 % (0/23), *p* = 0.02] than controls (Table 4). In addition, heterotopias were also more common in SUDC cases [21.7 % (15/69) vs. 0 % (0/23], *p* = 0.02). Gross asymmetry tended to be observed more frequently in SUDC cases: 43 % (9/69) as compared to 0 % (0/23) of controls (*p* = 0.11).

**Table 4:** Comparison of hippocampal and temporal lobe features between explained deaths and SUDC in the expanded San Diego dataset

	Explained		SUDC		*p* value
	*N* = 23		*N* = 69		
	Count	Percent	Count	Percent	
Focal granule cell bilamination	3	13.0	30	43.5	0.01
Gross malrotation	0	0	14	20.3	0.02
Heterotopia	0	0	15	21.7	0.02
Single ectopic granule cells in molecular layer	13	56.5	55	79.7	0.05
Clusters of ectopic granule cells in molecular layer	5	21.7	32	46.4	0.05
Gross asymmetry	0	0	9	42.8	0.11
Excess interstitial neurons	6	26.1	14	20.3	0.57
Anomalous formation of entorhinal cortex	1	4.3	7	10.1	0.67
Hyperconvolution	8	34.8	26	37.7	1.00
Glioneuronal heterotopia	0	0	3	4.3	1.00

The three most morphologically distinctive features identified in this cohort were focal granule cell bilamination, asymmetry, and malrotation. This combination, which we now term hippocampal malformation associated with sudden death (HMASD) was seen in 47.8 % (33/69) of SUDC cases with hippocampal sections compared to 13.3 % (3/23) of the explained cases (*p* = 0.003). All cases of gross asymmetry had one of the other defining features of HMASD: (1) focal granule cell bilamination (1/9); (2) malrotation (1/9) of the hippocampus; or (3) both asymmetry and malrotation (7/9). In the combined explained category (*n* = 23), no cases had gross malformations of the hippocampus, but three had focal granule cell bilamination. One explained case was part of the San Diego dataset, and was a 13-month-old healthy boy with a febrile seizure history who died suddenly during a sleep period, and was found prone; he had only a mild fever in the morning prior to death. The diagnosis of myocarditis was made based upon histologic examination of the heart at autopsy. The other two explained cases were from the BCH cohort and died of rapidly progressive lymphoma with CNS involvement and liver infarction due to torsion of a liver pedicle, respectively. The detailed findings in the hippocampus in HMASD are reported separately (Part II).

### The spectrum of neuropathology associated with sudden and unexpected death in young children

We detected neuropathologic disorders that were considered explained causes of death in cases with normal or no hippocampal sections. These included Williams syndrome (*n* = 1) with cortical dysplasia and serious congenital heart disease, viral meningitis with neurogenic pulmonary edema (*n* = 3), microcephaly, cerebral palsy, failure to thrive, and seizures (normal hippocampus) (*n* = 1); complex malformation of the brain (*n* = 1) with epilepsy and developmental delay, including medial and lateral temporal lobe, insula, and frontal lobes. There were 2 undetermined cases showing features of HMASD, which were initially classified as undetermined due to distinct findings of uncertain lethality: minimal brainstem encephalitis in one case and stable, untreated pilocytic astrocytoma of the cerebellum in the other. A final case in the San Diego cohort had the neuropathologic finding of severe hypoplasia of the arcuate nucleus of the ventral medullary surface, the putative homologue of the respiratory central chemosensitive zones in experimental animals. This has previously been observed in cases of sudden infant death syndrome, and is thought to be related to impaired central chemosensitivity and failure of response to asphyxia [14]. Because its precise relationship to sudden death is unknown, the death was classified as SUDC. No hippocampal sections were available for examination in this case.

## Discussion

We report findings related to sudden and unexpected death in young children from a dataset based upon physician or family self-referrals to a central registry. We found that hippocampal malformations are the leading neuropathologic abnormalities associated with sudden unexpected death in childhood. The most distinctive features seen in this series, defined as HMASD, were present in almost 50 % (40/83) of the total cases of sudden unexplained death in which hippocampal sections were available. These cases tended to be associated with fever around the time of death, and they were significantly more likely to have a personal and or family history of febrile seizures than explained cases [48.8 % (59/121) vs. 6.7 % (1/15), *p* < 0.001]. Thus, we confirmed and expanded the association of SUDC with a personal and/or family history of febrile seizures and/or HMASD, as in the initial cohort [7].

An additional key finding of this study is that approximately 50 % of the SUDC cases still remain unexplained after excluding those with HMASD. These SUDC deaths, unlike explained deaths, are associated with a sleep period (95 % of cases). This information provides a clue that sleep and/or transitions between sleep and waking are critical to the understanding of the etiology and pathogenesis of all SUDC cases. A proportion of these SUDC cases may have under sampled or unsampled HMASD. The detailed clinical and neuropathologic features of HMASD are reported separately.

An additional finding of this study is that the explained causes of death were not inherently preventable in the affected cases. Indeed, the available demographic information (e.g., term gestation, postnatal age at death, gender, and season of death) in this study is not significantly different between the explained and SUDC groups. While several of the infectious cases had a clinical prodrome, the symptoms were mild and non-specific, and did not herald a catastrophic illness or death. In addition, a significant portion of the SUDC cases had positive testing for common pathogenic, but not lethal organisms, raising the question of an underlying defect in immunological or inflammatory defense to hyper-acute microbial invasion that requires further research. Nevertheless, the types of organisms and the frequency of their presence were not substantially different from that in the explained cases. Two cases with potential cardiac channelopathies (one with a genetic variant of unknown clinical significance) were diagnosed in the three cases of the entire cohort tested with a cardiac gene panel. The case of William’s syndrome highlights that this entity is already well known to be associated with sudden death, but there are no specific measures known to prevent such death, other than careful monitoring and treatment of the underlying congenital heart disease, factors that were indeed under watch in our case [11].

This study has the advantage of a large sample of a very rare entity, allowing considerable statistical power to identify phenotypic subgroups. The causes of death were defined by strict centralized review by an experienced pediatric pathologist who had access to all available materials, including ancillary testing, the use of which showed considerable variation. The study’s retrospective nature is a drawback, as it is based on self-referral and thus potentially biased. This potential limitation is reflected in the high incidence of white families with high annual incomes and education levels, and in the high incidence of sudden unexplained (80 %) compared to population based studies of approximately 10 % [2–4]. In addition, the cohort includes no cases of homicide or suicide, preventing us from establishing the frequency of all causes of sudden and unexpected death, including unnatural causes. An additional limitation of this retrospective study is that no standard protocol is used by medical examiners across the country for tissue sectioning or sampling, or for ancillary testing in cases of sudden unexpected death. Moreover, tissue sampling in the different medical examiner’s offices in this study was generally limited. The establishment of standard protocols for the autopsy examination of cases of sudden unexpected death in infants and children will be a key step to permitting further and more in-depth research, and will require engagement, advocacy, and strong leadership by multiple stakeholders including forensic pathologists and neuropathologists and, importantly by patient advocates and bereaved parents.

In conclusion, we report that a substantial proportion of cases of sudden unexplained death in young children are associated with hippocampal maldevelopment with or without a febrile seizure phenotype. This study further emphasizes, however, that it is important not to overlook the fact that young children in the San Diego cohort also died of known causes of death (e.g., infections, cardiac channelopathies, non-febrile seizure disorders) that may possibly be preventable if detected in time. These explained deaths are not always preventable, however, mainly due to the lack of a clinical prodrome warning of impending death—with the premise that specific warning may have allowed time for intervention. Therefore, the national research agenda of sudden unexpected death in young children needs to be far-reaching. Diseases responsible for the unexplained deaths need to be identified. For known entities; e.g., infection, seizures, and cardiac arrhythmias, there is a need to identify or develop biomarkers of their potentially fatal risk. This study suggests limited availability and use of ancillary testing, particularly genetic and metabolic testing, in sudden unexpected death in childhood, although definitive conclusions on its use and utility cannot be drawn from the retrospective, non-population-based study. The approach to the eradication of all sudden death in young children should include a focus on research questions into the underlying mechanisms of known entities: do some children with a seemingly non-fulminant but fatal infection, for example, have an underlying immunological vulnerability? Research into the causes of SUDC needs to continue, by using standardized autopsies with systematic somatic, cardiac, and neuropathology reviews. The addition of state-of-the-art metabolic and genetic tools into death investigations will improve our understanding of these rare and elusive cases of sudden death.

## Key points

1. Hippocampal malformations are the leading neuropathologic abnormalities associated with sudden unexpected death in childhood.
2. The combination of malrotation, asymmetry, and/or focal bilamination, defined as hippocampal malformation associated with sudden death (HMASD), was present in almost 50 % of the total cases of sudden unexplained death in which hippocampal sections were available.
3. Cases with HMASD tended to be associated with male gender, prone discovery, fever around the time of death, and were significantly more likely to have a personal and or family history of febrile seizures than explained cases.
4. Approximately 50 % of cases of SUDC remain unexplained even after taking HMASD into account.

## References

[1] Krous H, Collins KA, Byard RW (2014). Sudden infant death syndrome (SIDS), sudden unexpected death in infancy (SUDI), and sudden unexplained death in childhood (SUDC). Forensic pathology of infancy and childhood.

[2] Helweg-Larsen K, Garde E (1993). Sudden natural death in childhood. A review of forensic autopsy protocols in cases of sudden death between the ages of one and five years, 1982–1991, with a special view to sudden unexplained death. Acta Paediatr.

[3] Southall DP, Stebbens V, Shinebourne EA (1987). Sudden and unexpected death between 1 and 5 years. Arch Dis Child.

[4] McGarvey CM, O’Regan M, Cryan J, Treacy A, Hamilton K, Devaney D (2012). Sudden unexplained death in childhood (1–4 years) in Ireland: an epidemiological profile and comparison with SIDS. Arch Dis Child.

[5] Byard RW (2010). Sudden death in the young.

[6] Kinney HC, Rognum TO, Nattie EE, Haddad GG, Hyma B, McEntire B (2012). Sudden and unexpected death in early life: proceedings of a symposium in honor of Dr. Henry F. Krous. Forensic Sci Med Pathol.

[7] Krous HF, Chadwick AE, Crandall L, Nadeau-Manning JM (2005). Sudden unexpected death in childhood: a report of 50 cases. Pediatr Dev Pathol.

[8] Kinney HC, Chadwick AE, Crandall LA, Grafe M, Armstrong DL, Kupsky WJ (2009). Sudden death, febrile seizures, and hippocampal and temporal lobe maldevelopment in toddlers: a new entity. Pediatr Dev Pathol.

[9] Kinney HC, Armstrong DL, Chadwick AE, Crandall LA, Hilbert C, Belliveau RA (2007). Sudden death in toddlers associated with developmental abnormalities of the hippocampus: a report of five cases. Pediatr Dev Pathol.

[10] Patterson JL, Carapetian SA, Hageman JR, Kelley KR (2013). Febrile seizures. Pediatr Ann.

[11] Duvernoy HM (2005). The human hippocampus: functional anatomy, vascularization, and serial sections with MRI.

[12] Krous HF, Chadwick AE, Miller DC, Crandall L, Kinney HC (2007). Sudden death in toddlers with viral meningitis, massive cerebral edema, and neurogenic pulmonary edema and hemorrhage: report of two cases. Pediatr Dev Pathol.

[13] Krous HF, Wahl C, Chadwick AE (2008). Sudden unexpected death in a toddler with Williams syndrome. Forensic Sci Med Pathol.

[14] Filiano JJ, Kinney HC (1992). Arcuate nucleus hypoplasia in the sudden infant death syndrome. J Neuropathol Exp Neurol.

